# Supplementary material for: Probing dark exciton navigation through a local strain landscape in a WSe2 monolayer
Source: Nat Commun. 2022 Jan 11;13:232. doi: 10.1038/s41467-021-27877-2 (PMC8752834; doi:10.1038/s41467-021-27877-2)
Supplement: Supplementary file 1 — Supplementary Information [file 41467_2021_27877_MOESM1_ESM.pdf]

Supplementary information to

"Probing dark exciton navigation through a local strain landscape in a WSe_2 monolayer"

Contents

1. Optical setup
2. Details of tapered fiber nanofabrication and FDTD simulation details
3. Rydberg states under strain
4. Charge tuning of WSe_2 before and after applying strain
5. Spectrum for streak camera data analysis
6. Details of mechanical modeling and reversibility of strain tuning
7. Numerical modeling of the drift-diffusion equation
8. Strain-tuning and charge-tuning spectra for $MoSe_2$

Supplementary Figure 1 – Optical Setup

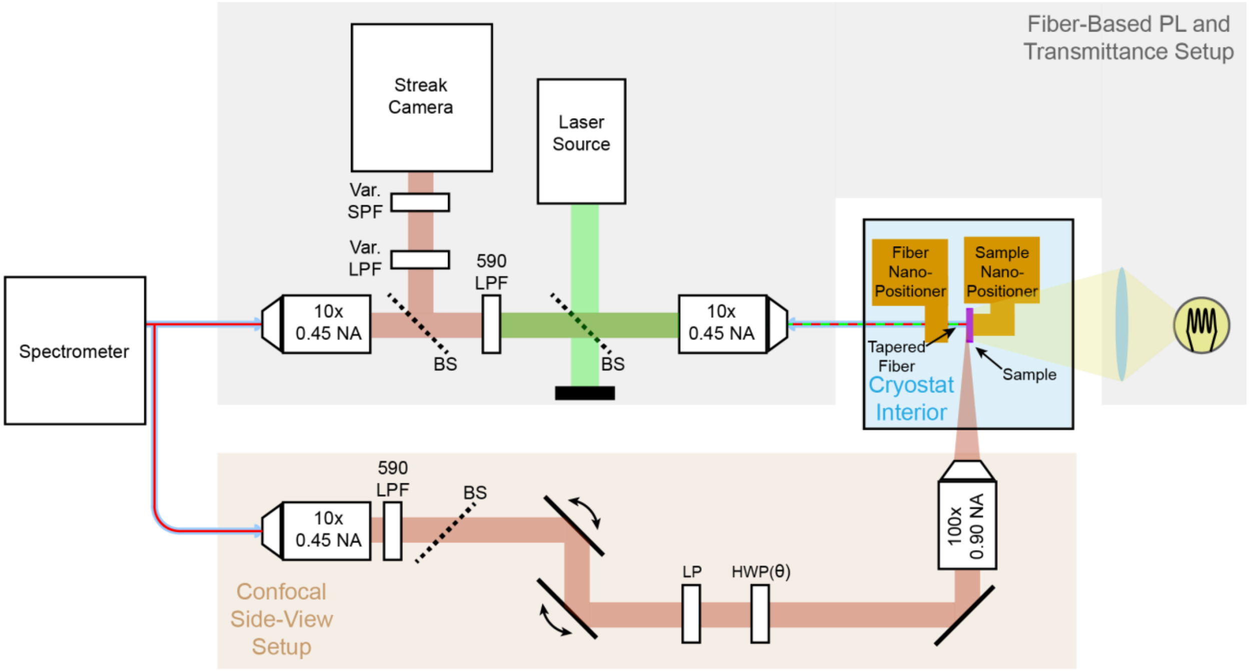

BS, Beamsplitter; LPF, Long-pass filter; SPF, short-pass filter; LP, linear polarizer; HWP, half-wave plate; NA, numerical aperature

The system is divided into three sections: the cryostat interior (blue), the fiber-based setup (gray), and the confocal side-view (brown). Within the cryostat are two piezoelectric nanopositioners upon which the fiber and sample are mounted and brought into contact. The fiber-based setup has a laser source (520 nm diode laser or 400 nm ultrafast pulsed laser, depending on the measurement). The laser beam passes through a beamsplitter (BS) before being coupled into the fiber which is routed into the cryostat. Alternatively, backside illumination by a broadband lamp may provide the excitation when probing transmittance. The collected signal (PL or transmittance) at the fiber facet is then routed back out of the cryostat along the same path where it is re-converted to a free-space optical beam. The beam passes through a 593 nm long-pass filter (Semrock FF593) to remove any laser light. A beamsplitter then allows us to either send the signal to a streak camera (with variable short-pass and long-pass filters (Var. SPF, Var. LPF)), or to a spectrometer. The confocal-side view collects PL from the sample while it is excited via the fiber. A 100x, 0.9 NA objective collects the light and passes it through a half-wave plate (HWP) on a rotating stage that rotates the polarization by θ, and linear polarizer (LP) to analyze the polarization. Two scanning mirrors allow us to collect only from the fiber facet. A 593 LPF eliminates laser light before sending the signal to the spectrometer.

Supplementary Figure 2 – Details of nanosculpted tapered fiber fabrication, FDTD simulation details and additional results

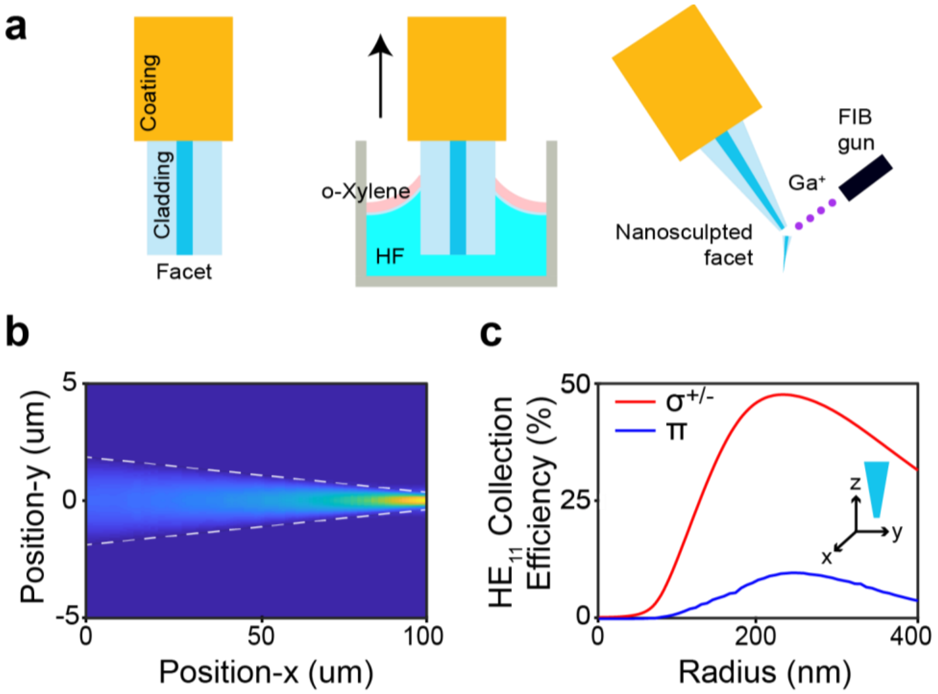

Nanosculpted tapered optical fibers are fabricated through a process illustrated in Figure S2a. Commercial near-infrared single-mode optical fibers (Thorlabs S630-HP) are first stripped of their buffer coating and cleaned. Afterwards, they are submerged in hydrofluoric acid (HF) to generate their taper profile [1]. A layer of o-Xylene sitting on top of the HF leads to the formation of a gradual taper via an oil-water interface meniscus that wicks up the length of the fiber. Drawing the fiber out of the HF solution at a constant rate enables control over the taper angle and leads to a self-terminating taper tip on the order of 10 nms. To deterministically cleave the taper tip, the self-terminated tapered fiber is mounted in a focused-ion beam (FIB) microscope. The tip diameter is then cut to the desired dimensions by etching away the excess fiber. Afterwards. a standard ionic-clean is carried out to remove potential residuary ions (Ga+) from the FIB processing. Post-fabrication fluorescence characterization of the nano-cleaved fiber confirms the absence of residual ions.

Eigenmode expansion simulations (via Lumerical) shown in Figure S2b depict how the fundamental fiber mode (HE11) transitions to the facet with negligible leakage. The large core-cladding contrast (~0.4, silica-vacuum at cryogenic temperatures) enables strong confinement of the optical mode. To optimize the collection efficiency of the fiber with respect to the facet radius, we perform finite-difference time-domain simulations of a dipole coupling into the fiber. Figure S2c shows the calculated HE11 coupling efficiency for an in-plane dipole ($\sigma^{+/-}$) and a z-polarized dipole (π) (coordinate reference shown in inset) as a function of facet radius. For larger radii, the efficiency increases before dropping due to the taper supporting higher-order modes. The coupling efficiency into higher order modes is not included due to the fact that they are not supported in the constant-cross section region of the fiber.

Supplementary Figure 3 – Rydberg states under strain

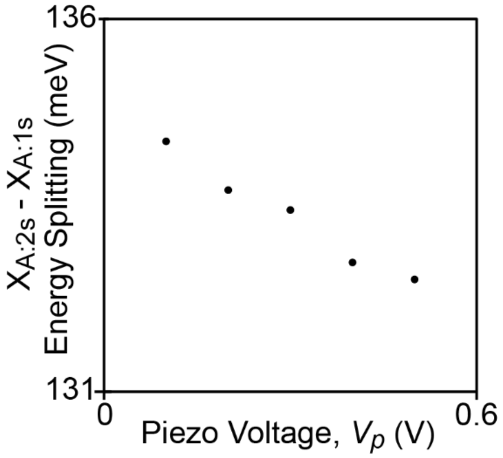

The splitting between the ground state ($X_{A:1s}$) and first excited Rydberg state ($X_{A:2s}$), obtained by fitting the transmittance spectra with Lorentzians, is a proxy for the exciton binding energy. This measure varies by only ~ 2 meV across the voltage range presented in Figure 1.

Supplementary Figure 4 – Charge tuning of WSe2 before and after applying strain

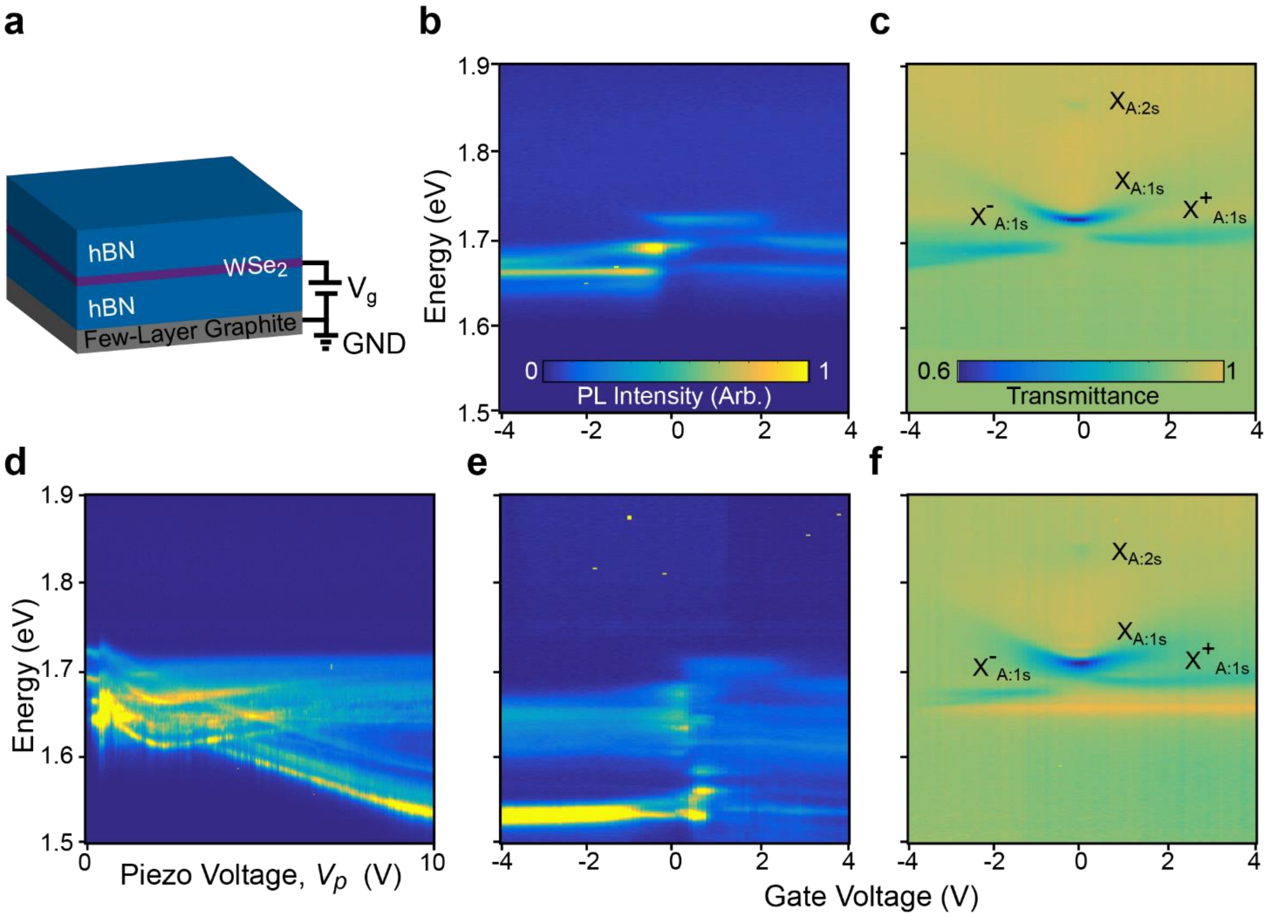

(a) Schematic of the gate configuration. (b) Charge-tuning PL and (c) transmittance spectra before applying strain (V_p = 0). (d) The strain-tuning PL spectra show the evolution from the low-strain to high-strain regimes. (e) Charge-tuning PL and (f) transmittance spectra after applying strain (V_p = 10 V). The A exciton ($X_{A:1s}$), its first Rydberg state ($X_{A:2s}$), and the positive (X^+) and negatively (X^-) charged excitons each shift by 35 meV in transmittance compared to the unstrained features.

Supplementary Figure 5 – Spectrum for streak camera data analysis

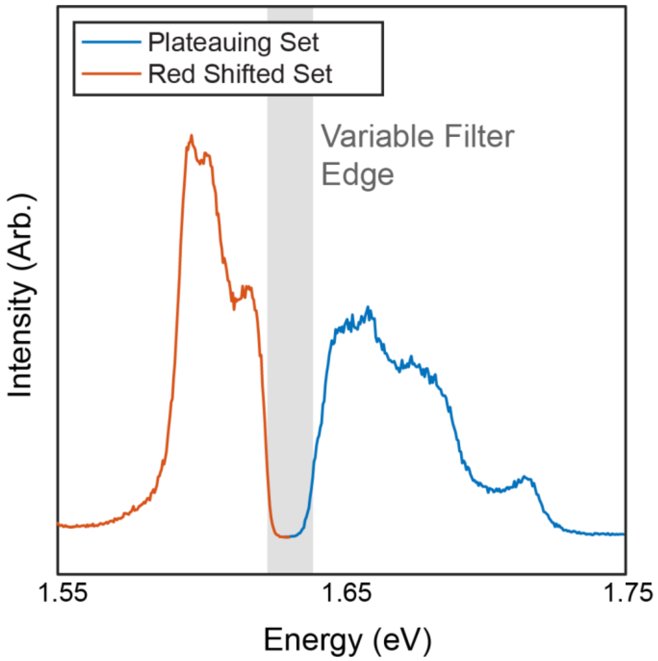

To observe the dynamics from the plateauing set and red shifted set independently, we filter the total spectrum using both a variable short-pass filter and long-pass filter to isolate the plateauing set only (blue) and red shifted set only (red). The integrated intensity from each of these sets were independently sent to the streak camera.

Supplementary Figure 6 – Details of mechanical modeling and reversibility of strain tuning

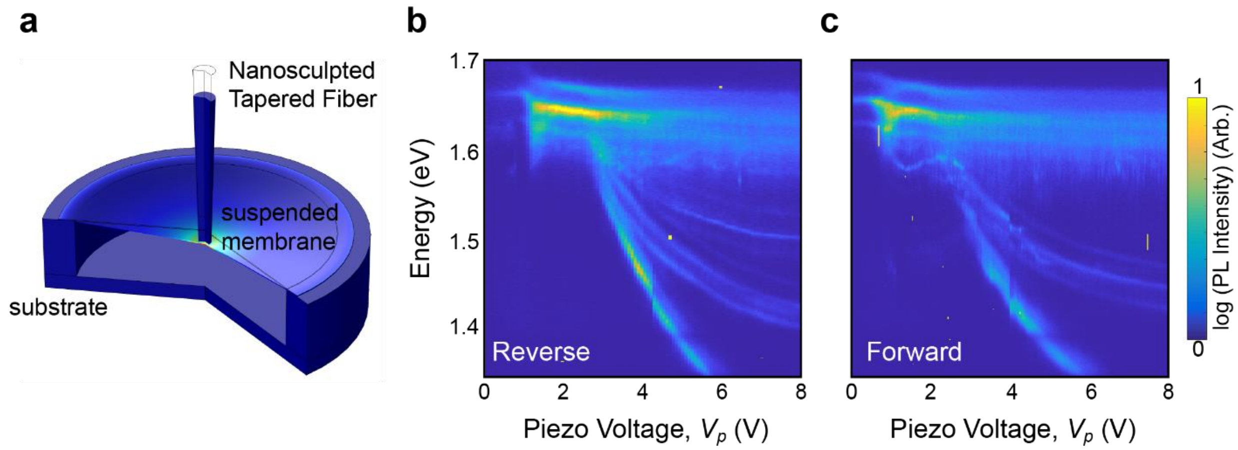

(a) COMSOL finite element method simulations are used to model strain in the hBN/WSe2/hBN heterostructure. Due to the thickness of the encapsulating hBN relative to WSe2 (hBN ~ 20nm >> WSe2 ~ 0.6 nm), we only use the mechanical properties of hBN in the simulation (young's modulus, density, Poisson's ratio). The model is composed of three parts: a nanosculpted tapered fiber, a suspended membrane, and a supporting substrate. The membrane is taken to be 20 nm thick, and a 240 nm radius facet is placed on the surface. To incorporate adhesion between the facet and heterostructure, the interface is treated as bonded. Note that in non-bonded simulations, the large strain at the fiber circumference shown in Figure 2b still exists. The membrane is clamped to the boundary of the substrate to eliminate slippage into the pit. This clamping is empirically accomplished through lithographically patterned Au/Cr electrodes deposited on all measured samples. The strain profiles plotted in Figure 2b are generated by translating the facet into the pit, and taking a line-cut of the strain profile 2nm below the center of the heterostructure.

(b,c) Show strain-tuning spectra showing the large tunability and reversibility achievable by this technique. Here, for spectra taken with the fiber moving (b) in reverse and (c) forwards, after several cycles, we continue to be able to extend a spectral feature by over 430 meV. We do not observe tearing during this process.

Supplementary Figure 7 – Numerical modeling of the drift-diffusion equation

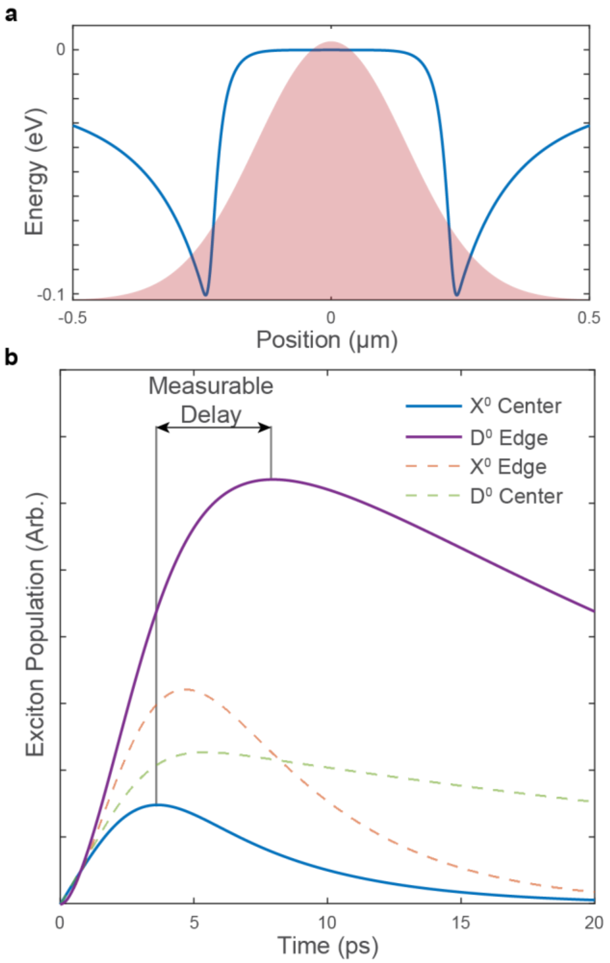

To verify our model of the dynamics in the system, we numerically solve a drift-diffusion partial differential equation for the exciton population *n*:

$$\frac{\partial n(x,t)}{\partial t} = D\nabla^2 n(x,t) - \mu\nabla \cdot \big(\boldsymbol{F}(\boldsymbol{x})n(x,t)\big) + I(x,t) - \frac{n(x,t)}{\tau}$$

The first term on the right-hand side is the diffusive term, with diffusivity *D*. The second is the drift term with mobility μ under the influence of a force field **F**, here derived from our strain potential $\boldsymbol{F} = -\nabla U = -\nabla(C\epsilon)$ for ε the strain and C the coefficient giving bandgap shift per strain. The third term is the laser intensity which populates the exciton states. The fourth is exciton decay during their lifetime τ. We take D = 1 cm^2/s [1], calculate the mobility using the Einstein relation at temperature T = 23 K, and take the exciton lifetimes from our streak camera measurements. (a) Depicts the function U(x) for a well depth of -0.1eV (blue curve) as well as the laser profile which sets the spatial profile of I(x,t). The temporal behavior of I(x,t) is a Gaussian with 2 ps width. (b) The time evolution of the exciton population at both the fiber center (center) and the well minimum (edge) is plotted for bright (X^0) and dark (D^0) excitons, with the only difference between the two being a lifetime of τ = 5 ps (bright) or τ = 48 ps (dark),

matching the data in Fig. 4. This model qualitatively captures the delay in population at the fiber edge observed in the time-resolved PL measurements.

Supplementary Figure 8 – Strain tuning and charge tuning of $MoSe_2$ spectrum

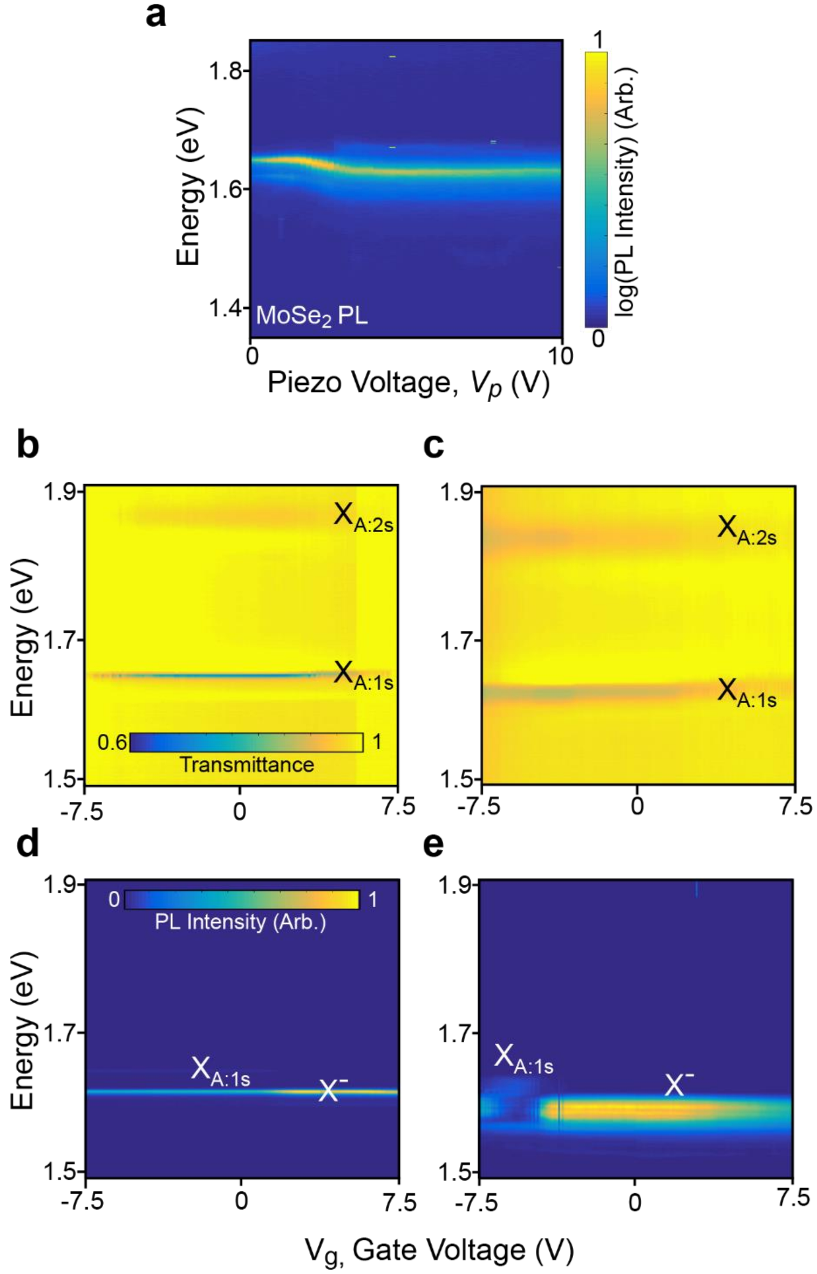

(a) Strain-tuning spectra of a hBN/$MoSe_2$/hBN/few-layer graphite device which shows only the plateauing features. (b-e) In analogy to Fig. S3, we do charge tuning on a $MoSe_2$ device and do not observe the same Stokes' shift between transmittance features and PL features, nor the presence of a red shifted family in any part of the charge tuning range.

References

1 Cordovilla Leon, D. F., Li, Z., Jang, S. W., Cheng, C.-H. & Deotare, P. B. Exciton transport in strained monolayer WSe2. *Applied Physics Letters* **113**, doi:10.1063/1.5063263 (2018).
